# Research on Compressive and Flexural Properties of Coal Gangue-Slag Geopolymer under Wetting-Drying Cycles and Analysis of Micro-Mechanism

**DOI:** 10.3390/polym13234160

**Published:** 2021-11-28

**Authors:** Xiaoyun Yang, Yan Zhang, Zhuhan Li, Minglei Wang

**Affiliations:** 1 College of Energy and Transportation Engineering, Inner Mongolia Agricultural University, Hohhot 010018, China; ycyangxiaoyun@emails.imau.edu.cn (X.Y.); wangshuoxin@emails.imau.edu.cn (M.W.); 2 Department of Civil Engineering, University of Victoria, Victoria, BC V8P 5C2, Canada; 3 School of Planning, University of Waterloo, Waterloo, ON N2L 3G1, Canada; z2246li@uwaterloo.ca

**Keywords:** coal gangue-slag geopolymer, wetting-drying cycles, compressive and flexural strength, micro mechanism

## Abstract

Coal gangue-slag geopolymer is a kind of environment-friendly material with excellent engineering performance and is formed from coal gangue and slag after excitation by an alkaline activator. In this study, three kinds of coal gangue-slag geopolymer were activated by different activators, and the compressive and flexural strengths of water and sulphate solutions in the wetting-drying (W-D) cycles were compared. The microscopic mechanism was analyzed by the XRD, the FTIR and the SEM. The following conclusions are drawn: The influence of W-D cycles on flexural strength was greater than compressive strength. The water migration and the recombination of geopolymers lead to the change of colour, as well as the reduction of flexural strength and compressive strength of geopolymers. The SH geopolymer had excellent anti-erosion ability in terms of flexural strength, and the reason for this was the recombination and polymerization reaction of geopolymer being weaker than the SS and the SSG. The corrosion resistance of the SS was reflected in the compressive strength, because its geopolymerization reaction was fierce, which produced more Na-rich C–N–A–S–H, N–A–S–H and C–A–S–H gels. Therefore, the compressive strength could still reach more than 39 MPa after 150 cycles. Sulfate solution could effectively control the reduction of compressive strength of the SH and the SS geopolymers during W-D cycles. The SSG had the worst corrosion resistance.

## 1. Introduction

A geopolymer is a three-dimensional inorganic polymer with high strength mechanical properties, physical properties, durability, and low carbon dioxide release [1,2,3,4]. Geopolymer can be formed quickly by silica aluminum materials (fly ash, slag, coal gangue, red mud, and other solid wastes) excited by alkaline solution [5]. Coal gangue is a by-product of coal, which has negative impacts on the surrounding environment, such as occupation, dust, collapse, landslide, soil erosion, toxic gas, heavy metal pollution, acid water pollution, and so on [6,7,8,9]. Coal gangue belongs to the coal-measure kaolinite, and its main chemical composition is SiO_2_ and Al_2_O_3_ [10]. After bleaching, high-temperature calcination, and grinding, coal gangue will be transformed into high-purity kaolin, and it can be transformed into metakaolin when the calcination temperature is controlled in the low-temperature calcination zone, 500–900 °C [11]. Metakaolin is a good raw material for geopolymers [12]. Compared with cement, calcined coal gangue has become the most prospective industrial by-product due to its low carbon footprint [13]. However, geopolymers prepared only with coal gangue as raw materials have a long hardening time and low strength after alkali activation, which limits their wide application in engineering. Ground granulated blast furnace slag (GGBFS) is a highly active powder, and alkali-activated slag (AAS) is a new type of gelling agent with good mechanical properties and excellent chemical durability [14]. GGBFS can also form high-strength geopolymers after being excited by alkalis [15,16,17], but its price is relatively expensive, and the large-scale use of slag will increase the cost of geopolymer preparation. The geopolymer produced by the combination of coal gangue and GGBFS can produce high-strength geopolymer in a short time [18,19], and the use of coal gangue as the main material can reduce the cost of preparing geopolymers.

In recent years, extreme weather has occurred frequently. Geopolymers will shrink cracks or decrease strength due to water evaporation or material transformation in high temperature or humid weather [20]. There are few studies available on coal gangue-based geopolymers in dry and wet cycle environments. Whether coal gangue-based geopolymers can adapt to extreme weather conditions has become a problem that must be considered for its wide promotion in engineering. Therefore, the compressive and flexural strength performance of coal gangue-slag geopolymer under dry and wet environments was studied. Water and sulphate resistance were evaluated by appearance, residual compressive strength, and corrosion resistance coefficient (K_f_), and the microscopic mechanism was analyzed by X-ray diffractometry (XRD), fourier transform infrared radiation (FTIR), and scanning electron microscopy (SEM).

## 2. Raw Materials and Methods

**Figure 1:**
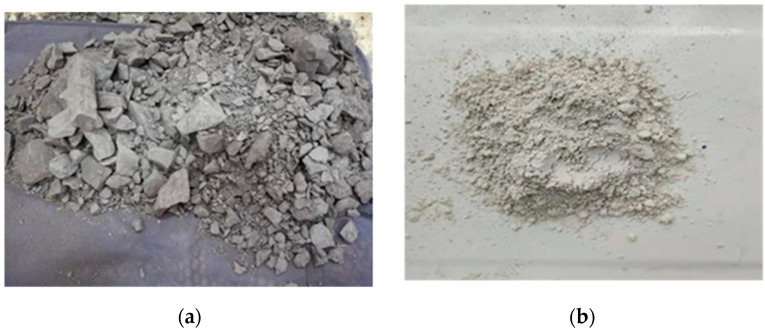
Raw materials. (**a**) Raw coal gangue; (**b**) Slag.

Coal gangue and slag powder were obtained from Shanxi Shuguang Coal Coke Group Co.,Ltd. and Hejin city Huaxinyuan Iron and Steel Co.,Ltd., Hejin City, Shanxi Province, China. Activated coal gangue powder is a good raw material for geopolymers. The activation step was used to manually smash the coal gangue into a muffle furnace. Then, it was calcinated at 700 °C for 2 h, and finally crushed to less than 45 μm with an ore crusher. The grade of slag is S95. Figure 1 shows the raw materials.

In this experiment, three types of geopolymer were used for a comparative analysis of wetting and drying (W-D) cycles. The three geopolymers are represented by SH, SS, and SSG, respectively. The SH geopolymer was formed by the excitation of 2 mol/L NaOH. The SS geopolymer activator was Na_2_SiO_3_ + NaOH (18% content, modulus 0.8). The SSG geopolymer activator was desulfurized gypsum + NaOH + Na_2_SiO_3_ (6% desulfurized gypsum, the content of NaOH + Na_2_SiO_3_ was 16%, the modulus was 0.6). The preparation of W-D cycle specimens included the following steps. The prepared activator and water were stirred in the magnetic mixer for 30 min. Then, they were poured into the mixer (NJ-160A cement paste mixer, Wuxi Xiyi Building Material Instrument Factory, Wuxi City, China) together with the coal gangue and slag powder mixture. After mixing, the slurry was poured into the triple mould (40 × 40 × 160 mm^3^) according to the standard method, and 60 s shaking was performed on the vibrating table. After that, it was placed in the standard curing box (Humidity is above 95%, the temperature is 20 ± 2 °C) for 24 h, the mould was removed, and curing continued for 28 days [21].

The W-D cycles were divided into the water W-D cycles (deionized water) and the sulphate solution (5% Na_2_SiO_4_) W-D cycles. The strength test result was the average strength of 6 specimens. The total time of each W-D cycle was (24 ± 2) h with following segments: The test piece was soaked in the solution for (15 ± 0.5) h, air-dried for 30 min, dried in an oven for 6 h (the temperature of the oven was (80 ± 5) °C), and cooled for 2 h [22]. In the first 75 cycles, the flexural and compressive strength of the test pieces were tested every 15 times. The strength was measured by the automatic bending and compression instrument (WYA-300B, Wuxi Xiyi Building Materials Instrument Factory, Wuxi City, China). After 75 times, the test pieces were tested every 25 times until the end of 150 times. Test termination situation is following: The mass loss rate of W-D cycles reaches 5%. The corrosion resistance coefficient of compressive strength reaches 75%. The calculation method is shown in Equation (1).

(1) $$K_{f}=\frac{f_{\mathrm{cn}}}{f_{c0}}\times 100$$

where K_f_ denotes the corrosion resistance coefficient of compressive strength (%); f_cn_ the compressive strength value of specimens corroded by sulphate after N dry-wet cycles (MPa), accurate to 0.1 MPa; f_co_ the compressive strength value of standard curing specimens at the same age (MPa), accurate to 0.1 MPa.

After completing the strength test, the specimens were crushed and soaked in ethanol for 24 h to terminate the hydration reaction. Then, the powder was dried, and the XRD (D8 Advance, Bruker AXS, Karlsruhe, Germany, the scanning angle is 5°–90°, 8° per minute), the SEM (Zeiss Sigma 500, Zeiss, Oberkochen, Germany), and the FTIR ( Nicolet iS5, Thermo Fischer Scientific Inc., Waltham, MA, USA) methods were used to analyze the microscopic mechanism.

## 3. The Appearance of the Specimen

**Figure 2:**
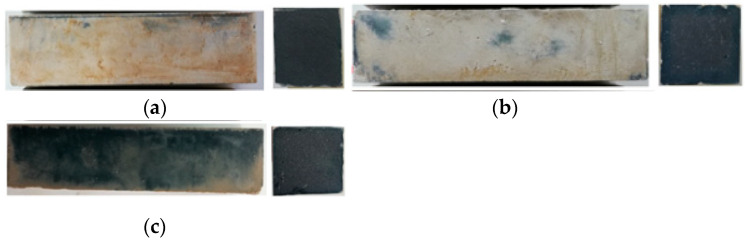
Surface and cross-sectional views of the specimens of standard curing after 28 days. (**a**) SH geopolymer (**b**) SS geopolymer (**c**) SSG geopolymer.

After the W-D cycles, the surface of the test pieces changed in different degrees, and the integrity of the test pieces can be evaluated according to the appearance change. The side and cross-sectional views of the three geopolymers cured for 28 days under standard conditions are shown in Figure 2. The surface of the specimens after 28 days of curing was smooth, the surface was white-blue and dense, and the uniform blue geopolymer was formed on the cross-section.

### 3.1. W-D Cycles of Water

**Figure 3:**
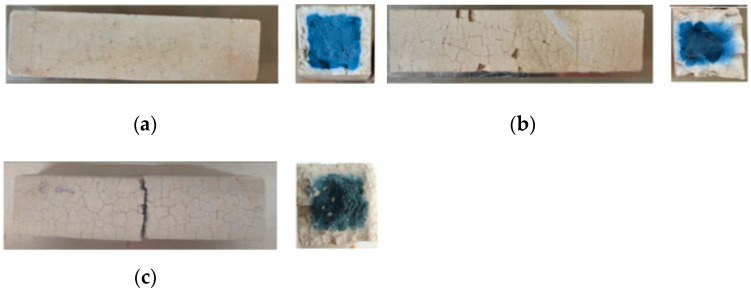
The surface of specimens after water W-D cycles. (**a**) SH 150 cycles (**b**) SS 150 cycles(**c**) SSG 20 cycles.nsed than the SH, and the colour of the blue substance was relatively lighter. Some of the SSG specimens began to break into two sections along the cracks at 20 cycles, and this section also showed that the surrounding blue geopolymer turned white.

Figure 3 illustrates the surface of the water W-D cycles test pieces. Due to the influence of water cycles, cracks grew on the surface of the specimens, forming grid-like cracks. Few cracks formed on the surface of the SH geopolymer, while SS geopolymer had more cracks on the surface, even some surfaces dropped, and edges were missing. It can be seen from the cross-sectional view that the colour of the geopolymer gradually changed from blue to white after the water W-D cycles, and polycondensation from the outside to the inside occured.

**Figure 4:**
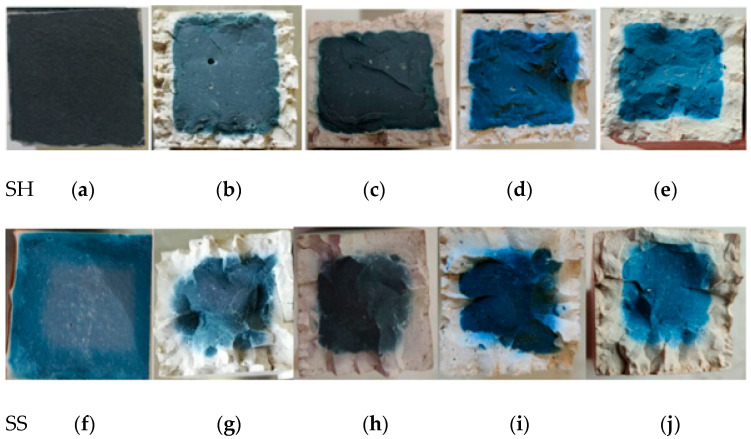
Cross-sectional views of the SH and the SS after water W-D cycles. (**a**) SH 0 cycles (**b**) SH 15 cycles (**c**) SH 30 cycles (**d**) SH 60 cycles (**e**) SH 100 cycles (**f**) SS 0 cycles (**g**) SS 15 cycles (**h**) SS 30 cycles (**i**) SS 60 cycles (**j**) SS 100 cycles.

To observe the polycondensation phenomenon in detail, take the SH and the SS as an example, presented in Figure 4. The surrounding section changed from the original blue geopolymer to white, the white geopolymer evolved from the periphery to the center, and the blue geopolymer in the center began to fade in colour.

### 3.2. W-D Cycles of Sulphate

**Figure 5:**
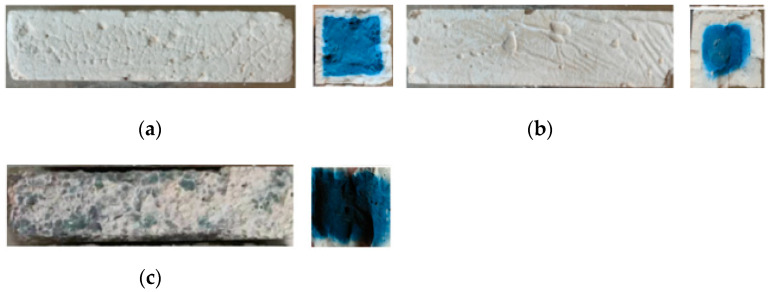
Surface and cross-sectional view of the specimens after sulphate W-D cycles. (**a**) SH 150 cycles (**b**) SS 150 cycles (**c**) SSG 3 cycles.

All the specimens were corroded, a layer of white sulphate was deposited on the surface, grid cracks appeared, and the external surface peeled off after the sulphate W-D cycles (Figure 5). The blue polycondensation of the SS geopolymer was more obvious, and the change from blue to white around the SH geopolymer was slower. After three cycles, the white geopolymer on the surface of the SSG sample fell off seriously, and the average mass loss rate was 27.3% > 5%. Meanwhile, the entire SSG specimen was crisp, and the flexural strength and compressive strength were only 0.5 MPa and 9.7 MPa (K_f_ < 75%), respectively. Therefore, the sulphate W-D cycle test of the SSG specimen was terminated, which proved that the SSG geopolymer was not resistant to sulphate corrosion.

**Figure 6:**
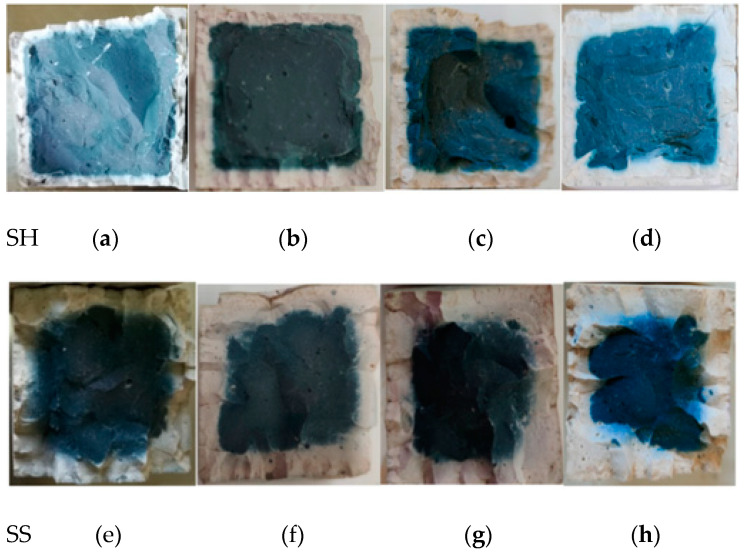
Cross-section of SH and SS geopolymer sulphate W-D cycles. (**a**) SH 15 cycles (**b**) SH 30 cycles (**c**) SH 60 cycles (**d**) SH 100 cycles (**e**) SS 15 cycles (**f**) SS 30 cycles (**g**) SS 60 cycles (**h**) SS 100 cycles.

The SH and SS geopolymer are taken as an example to describe the sulphate corrosion process (Figure 6). In the sulphate W-D cycles, a similar situation of the water cycle also occurred, with white geopolymers appearing on the edges and blue geopolymers only in the center of the specimen. Moreover, as the number of cycles increased, the white ceramic materials gradually increased and became denser.

## 4. Compressive and Flexural Strength Test Results

### 4.1. W-D Cycles of Water

**Figure 7:**
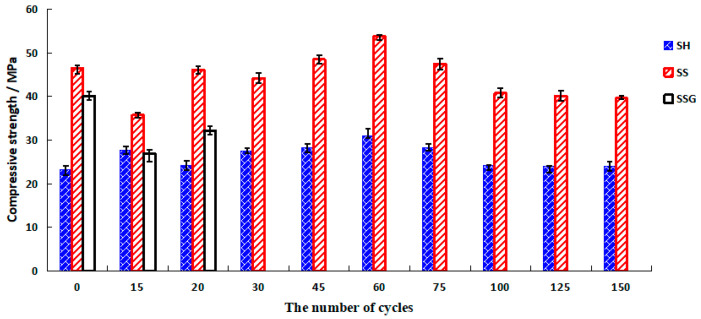
Compressive strength diagram of water W-D cycles.

Figure 7 presents the compressive strength of the water W-D cycles. The SH and the SS showed an increasing trend in the first 60 cycles, before decreasing after 60 cycles. The peak values were 31 MPa and 53.8 MPa, respectively, and the final values were stable at 24 MPa and 39.6 MPa, respectively. Some of the SSG specimens were broken into two pieces along the crack at 20 cycles, and the final compressive strength value was 32.2 MPa.

**Figure 8:**
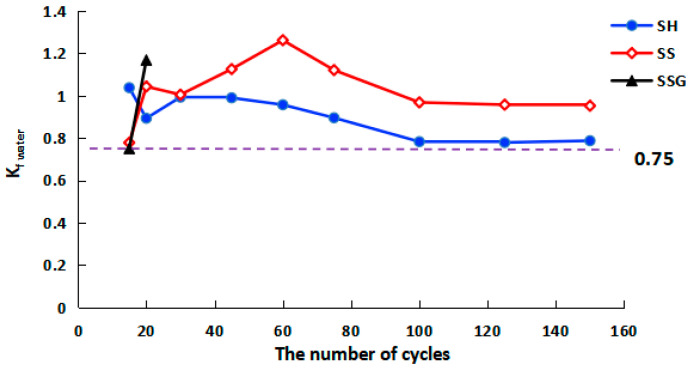
K_f water_ curve of compressive strength in water W-D cycles.

The curves of the corrosion resistance coefficient after the water W-D cycles (K_f water_) are illustrated in Figure 8. The K_f water_ of the SH and the SS geopolymers were both above 0.75. The SS geopolymer had the best resistance to water circulation, followed by the SH, and the SSG was the worst. In 20–75 cycles, the K_f water_ of the SS geopolymer was greater than 1, which proved that the water W-D cycles were beneficial to improve the compressive strength of SS geopolymer in the early cycles.

**Figure 9:**
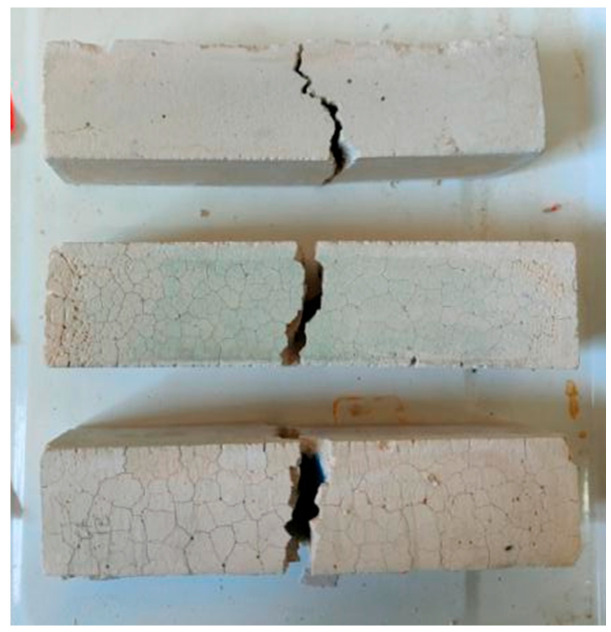
Specimen damaged in flexural test.

**Figure 10:**
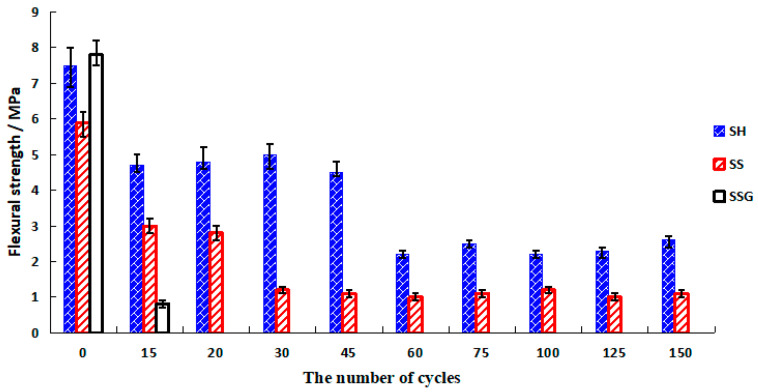
Flexural strength diagram of water W-D cycles.

Figure 9 shows specimens destroyed along cracks in the flexural test. The flexural strength of water W-D cycles is shown in Figure 10. Flexural strength of the SH increased during the first 30 cycles, then decreased rapidly, and finally stabilized at 2.6 MPa. The SS flexural strength decreased after 15 cycles and stabilized at about 1 MPa after 30 cycles. The SSG had been broken after 20 cycles. Therefore, the flexural resistance of the SH geopolymer was better than that of the SS geopolymer in water cycles, which was related to its fewer fractures.

**Figure 11:**
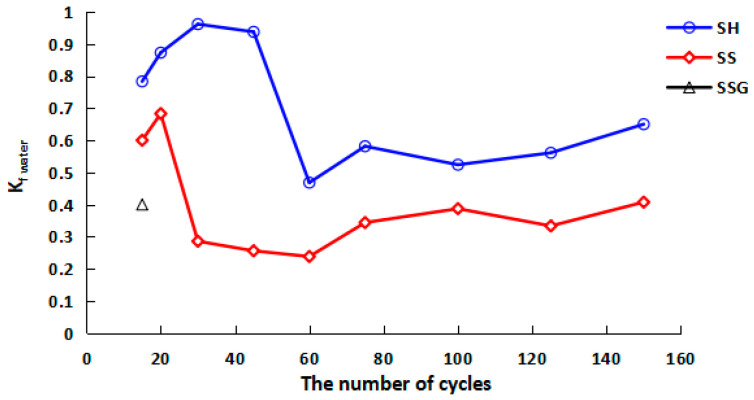
K_f water_ curve of flexural strength in the water W-D cycles.

The specimens under standard curing condition also showed the rule that the flexural strength decreased with the increase of time after 28 days, which proved that the specimens were constantly hardened in the curing process (the compressive strength remained at a high level while the flexural strength decreased) and transformed into brittle materials, and this change process is similar to concrete. The average final flexural strength values of the SH, SS, and SSG specimens without W-D cycles were 4 MPa, 2.7 MPa, and 2.2 MPa, respectively, which were 3.5 MPa, 3.2 MPa, and 5.6 MPa lower than the 28-day flexural strength. Figure 11 describes the flexural strength K_f water_ water cycles curve. The K_f water_ values of SH were above 0.8 in the early cycles, decreased significantly after 45 cycles, and finally stabilized between 0.5 and 0.6. The K_f water_ values of SS dropped faster in the water W-D cycles, and finally stabilized between 0.3 and 0.4. The K_f water_ of SSG was only 0.4 at 20 cycles. These results proved that the cracks and the material changes of the W-D cycles had an impact on the specimens.

### 4.2. W-D Cycles of Sulphate

**Figure 12:**
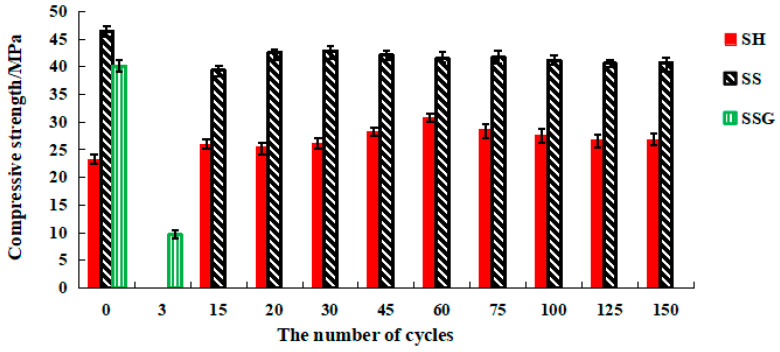
The compressive strength diagram of sulphate W-D cycles.

Figure 12 shows that the compressive strength of the SH specimens gradually increased before 60 cycles, reaching a maximum of 30.9 MPa, then gradually decreased as the number of cycles increased, and finally stabilized at 26.7 MPa. The compressive strength value of the SS specimen increased during the first 30 cycles, gradually decreased after more than 30 cycles, and finally stabilized at 40.8 MPa. The SSG fractured after three cycles, and the compressive strength was only 9.7 MPa.

**Figure 13:**
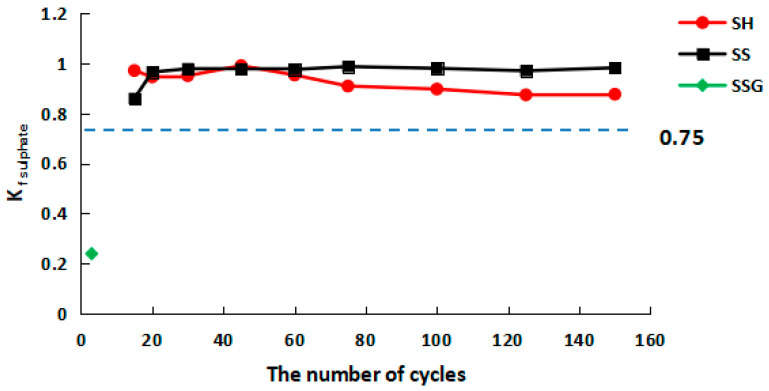
K_f sulphate_ curve of compressive strength in sulphate W-D cycles.

In Figure 13, the sulphate corrosion resistance coefficient values (K_f sulphate_) of the SH and the SS geopolymers were greater than 0.75, the K_f sulphate_ values of the SH geopolymers were above 0.87, and the K_f sulphate_ values of SS geopolymers were above 0.96. The SS had the best resistance to sulphate corrosion, followed by the SH, and the SSG was the worst.

**Figure 14:**
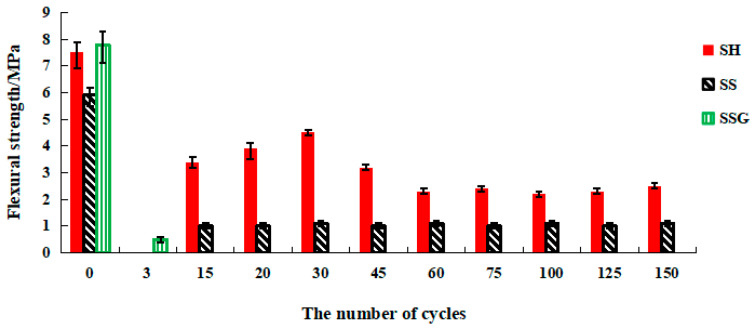
Flexural strength diagram of sulphate W-D cycles.

In Figure 14, the SH geopolymer shows an upward trend between 15 and 30 cycles, which then drops sharply, basically stabilizing between 2.2 and 2.5 MPa. The flexural performance of the SH geopolymer was better than the SS and the SSG geopolymer, mainly because there were fewer cracks during the cycles. The flexural strength of SSG dropped to 0.5 MPa in the third cycle.

**Figure 15:**
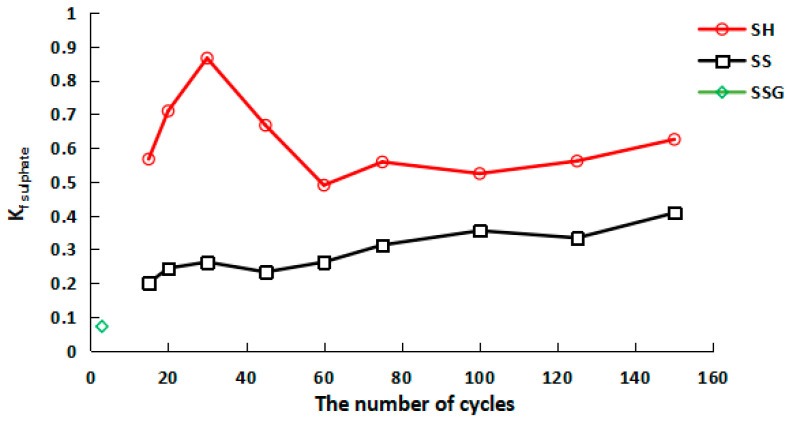
K_f sulphate_ curve of flexural strength in sulphate W-D cycles.

The change trend of K_f sulphate_ in the sulfate cycle is described in Figure 15. The sulphate W-D cycles also showed the effect of cracks and hardening on its flexural strength. SH fluctuated greatly in the early stage, showing a trend of first increasing and then decreasing. After 60 cycles, K_f sulphate_ remained between 0.5 and 0.6. However, SS maintained a relatively stable trend, fluctuating between 0.2 and 0.4. The K_f sulphate_ of SSG was only 0.07 after three cycles.

### 4.3. Comparative Analysis of the W-D Cycles of Water and Sulphate Solution

The differences in appearance, compressive and flexural strength, and K_f_ between the water cycles and the sulphate cycles are given in Table 1. The SH geopolymer did not easily form cracks in the W-D cycles, so its flexural strength was higher. The K_f_ value of the SH geopolymer was lower than that of the SS geopolymer. The SS geopolymer formed cracks more easily, the flexural strength decreased faster, and because of the shallow crack on the surface, it has little effect on the compressive strength. In conclusion, in terms of erosion resistance, the SH geopolymer was superior in terms of flexural strength, while the SS geopolymer was dominant in terms of compressive strength. The SSG had the weakest erosion resistance. The W-D cycles of the sulphate solution were beneficial to inhibit the decrease of the compressive strength of the SH and the SS geopolymers. However, the resistance of the SSG to sulphate erosion was weaker than that of water erosion.

## 5. Micro Mechanism Analysis

### 5.1. XRD

**Figure 16:**
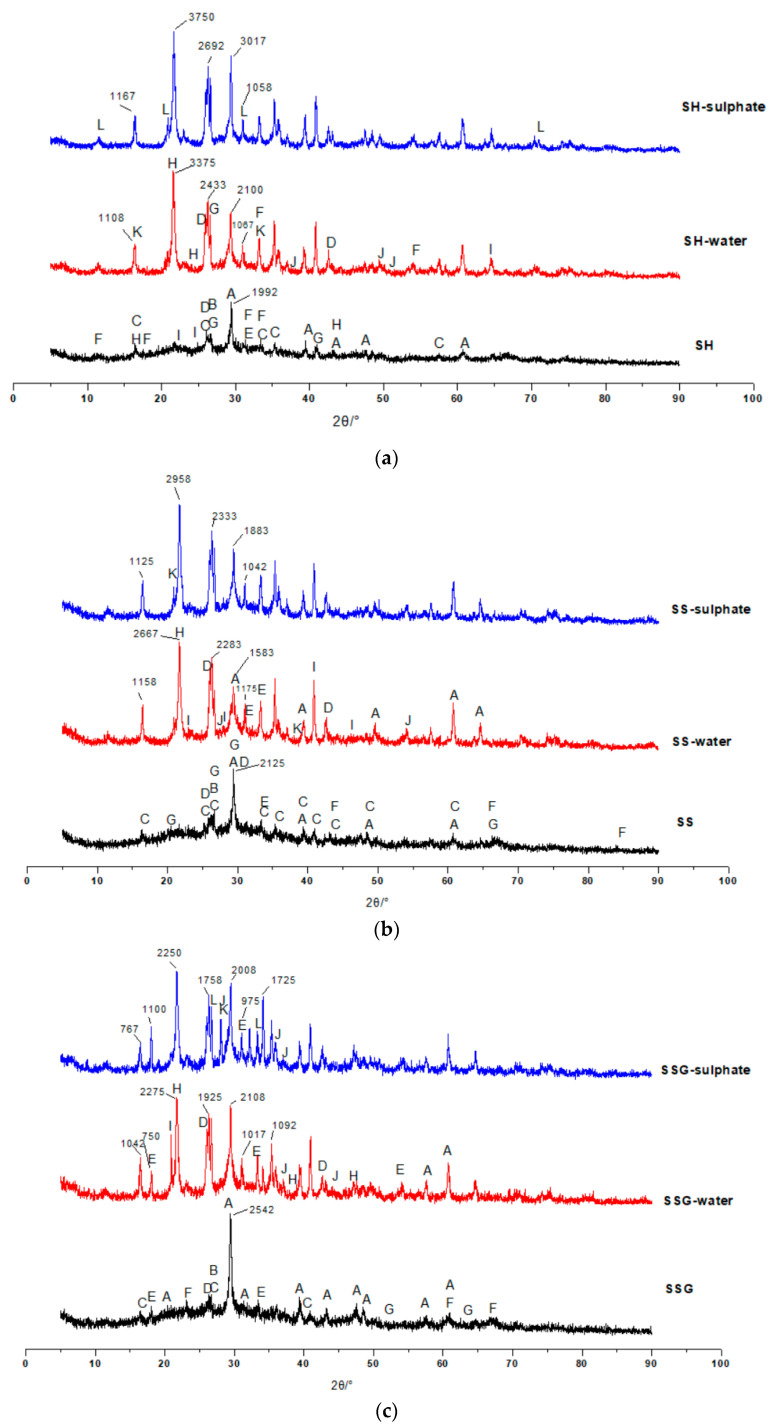
XRD pattern of coal gangue-slag geopolymer. (**a**) XRD pattern of the SH geopolymer. A-Calcite B-SiO_2_ C-Sillimanite D-Analcime E-Gehlenite F-Zoisite G-Kyanite H-Lisetite I-Nepheline J-Labradorite K-Calcium natrium aluminum silicon oxide L-Gypsum. (**b**) XRD pattern of the SS geopolymer. A-Calcite B-SiO_2_ C-Sillimanite D-Analcime E-Zoisite F-Nepheline G-Kyanite H-Lisetite I-Labradorite J-Na-rich Anorthite K-Gypsum. (**c**) XRD pattern of the SSG geopolymer. A-Calcite B-SiO_2_ C-Sillimanite D-Analcime E-Zoisite F-Nepheline G-Labradorite H-Lisetite I-Gypsum J-Anorthite K-Ca-rich Albite L-Kyanite.

The main hydration products of geopolymers are C–(A)–S–H, N–A–S–H and C–N–A–S–H gels. According to the XRD patterns in Figure 16, the three geopolymers were composed of calcite, silicon dioxide, sillimanite, analcime, gehlenite, zoisite, kyanite, nepheline, and other N–A–S–H and C–A–S–H gels. Among these minerals, nepheline, sillimanite, kyanite, zoisite, and analcime are blue and green, so the colour of the specimens was blue-green (corresponding to Figure 2). After the W-D cycles, the disordered N–A–S–H transforms into a crystalline zeolite phase structure due to drying at a high temperature of 80 °C [23]. From Figure 16, it is clear that all three geopolymers had other characteristic peaks. There were obvious characteristic peaks of Lisetite (CaNa_2_Al_4_Si_4_O_16_), Labradorite (Na_0.45_Ca_0.55_Al_1.5_Si_2.5_O_8_), Na-rich Anorthite, Ca-rich Albite, and other C–N–A–S–H gels between 15° and 45°. The characteristic peaks of other minerals were also stronger than those of the samples without the W-D cycles, indicating that the W-D cycles promoted the formation of C–N–A–S–H gel, increased N–A–S–H and C-S-A-H gels, and gypsum products were also present. C–A–S–H and C–N–A–S–H gels are denser and stronger than N–A–S–H gel. The colour of C–N–A–S–H is white, so the specimens had the appearance of white and dense ceramic-like materials, which correspond to the transition from blue substances to white substances in the W-D cycles in Figure 3, Figure 4, Figure 5 and Figure 6. Meanwhile, this also confirmed the conclusion that the specimen gradually hardened, and the W-D cycles had little influence on the compressive strength of the specimen but a great influence on the flexural strength proposed in Section 4. However, the migration of water and the reorganization of geopolymers formed from the outside to the inside caused cracks on the surface of the specimens and formed a sandwich pattern, resulting in irreversible strength loss.

The products of the sample subjected to the W-D cycles of sulphate solution were similar to those of the water cycle. The peak of calcite after W-D cycles in SH was higher than that of uncycled (Figure 16a), demonstrating that the carbonization strength had increased and Ca^2+^ had not been fully added to the geopolymerization reaction, which corresponded to the appearance of small cracks and small white borders (see Figure 4 and Figure 6). However, the calcite of the SS and the SSG decreased in the W-D cycles compared with that without the cycles (Figure 16b,c), indicating that the W-D cycles reduced the carbonization of the sample, and the excess Ca^2+^ participated in the reaction to generate the C-S-A-H gel and C–N–A–S–H gel. Lisetite (C–N–A–S–H gel) peaks were higher in both SH and SS sulphate cycles than in the water cycle, demonstrating that sulphate promoted C–N–A–S–H gel formation, which was consistent with K_f sulphate_ remaining at a higher level and with less reduction in compressive strength values in sulphate. Desulphurized gypsum was present in the SSG activators, thus introducing Ca^2+^. With a large amount of Ca^2+^ involved in the reaction, more C–A–S–H gel and Ca-rich C–N–A–S–H gel appeared in W-D cycles. In terms of specimen surface contact with the outside world, the severe reaction started from the surface, while the substance in the center of the specimen had no time to react, leading to the peeling from the skin and center. In the sulphate cycles, there were more characteristic peaks of the cementitious material, proving the severe reaction, which corresponded to only three cycles in the sulphate cycles.

### 5.2. FTIR

**Figure 17:**
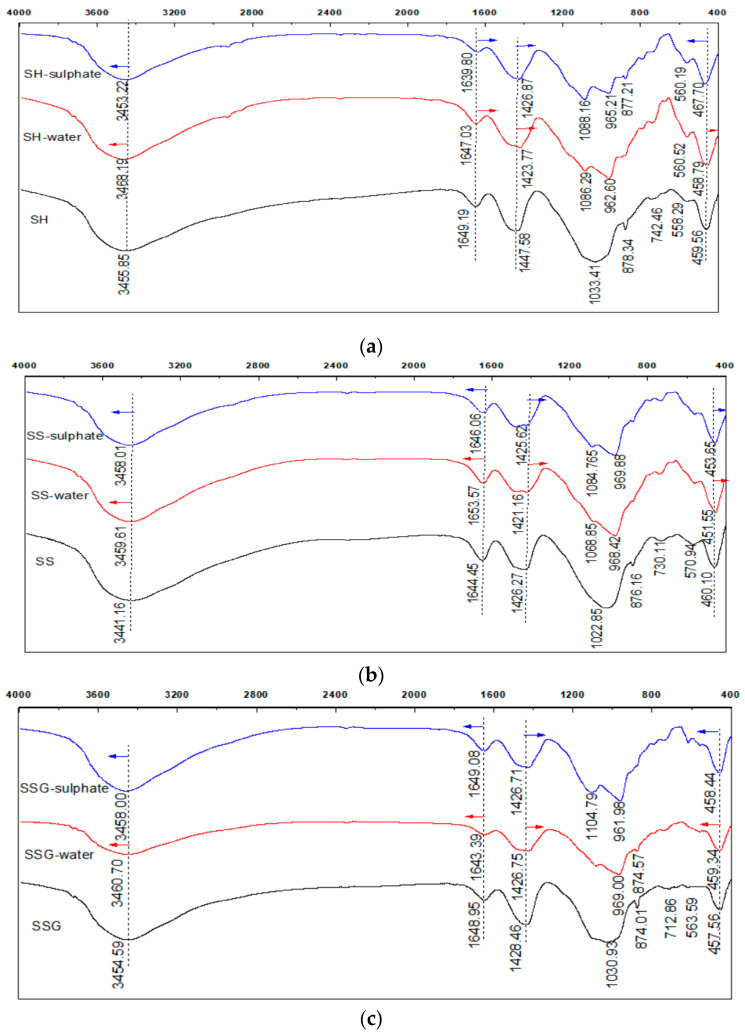
FIRT spectrum of geopolymer. (**a**) FIRT spectrum of the SH geopolymer. (**b**) FIRT spectrum of the SS geopolymer. (**c**) FIRT spectrum of the SSG geopolymer.

In Figure 17, the main peak positions of the characteristic spectral lines of the three geopolymers were the same, indicating that they had similar material compositions. The adsorption zones were located at 3440–3460 cm^−1^ and 1644–1649 cm^−1^, which were caused by the OH^−^ tensile vibration and the H–O–H bending vibration caused by the hydration water on the surface of the geopolymer, respectively [24,25]. After the W-D cycles, the intensity of this area decreased and moved to high frequency, which proved that the W-D cycles caused part of the adsorbed water to escape and the crystal structure to collapse. The wavelength of 1426–1447 cm^−1^ was the O–C–O tensile vibration peak, which was due to the reaction of CO_2_ in the air with alkali to form carbonate [26]. After the W-D cycles, the peak strength weakened and moved to the low frequency, which that the group was unstable and the calcium carbonate content decreased, and the W-D cycles inhibited the carbonization reaction. The characteristic strong band at 900–1200 cm^−1^ reflected the formation of geopolymers, which was related to the asymmetric stretching vibration of T–O–Si (T=Al, Si) formed by TO4 [27,28]. The shift of the frequency band to a lower frequency in this region proved that the polymerization reaction was violent [29]. Therefore, the rate of polymerization of geopolymers in the order of high to low effect is the SS, the SSG, and the SH. In the W-D cycles, the 900–1200 cm^−1^ region formed two characteristic spectral lines, which proved the vibration and reunion of the T–O–Si bond, and a new phase was formed. Moreover, 1068–1104 cm^−1^ is the typical position of the main band in the N–A–S–H gel, and 961–969 cm^−1^ represents the C–A–S–H gel [30]. Therefore, the geopolymer of coal gangue-slag under standard curing conditions was mainly the N–A–S–H gelation, which reflected the geopolymer characteristics of the low calcium system. After the W-D cycles, the C–A–S–H and the C–N–A–S–H gels were generated at high temperature and water loss, which indicated the characteristics of high calcium system geopolymer or Na-rich geopolymer formed after the slag was excited. Further, the wave number moved to a higher wavenumber, illustrating that the group was more stable, and the conversion from amorphous cementation to stable zeolite. In the sulphate solution, because the Na_2_SO_4_ solution provided an alkaline environment for the geopolymer, the wavenumber moved to a higher wavenumber, and the crystallinity was high. Therefore, the compressive strength of the geopolymer in the sulphate solution was higher than the compressive strength of the water. The vibrations around 558–742 cm^−1^ were asymmetric and symmetric vibrations of the Si–O–T bond of the Geopolymer gel of Al4 and SiO_4_ tetrahedra [31]. Moreover, 451–467 cm^−1^ was the bending vibration of Si–O–Si and O–Si–O [27,32].

### 5.3. SEM

**Figure 18:**
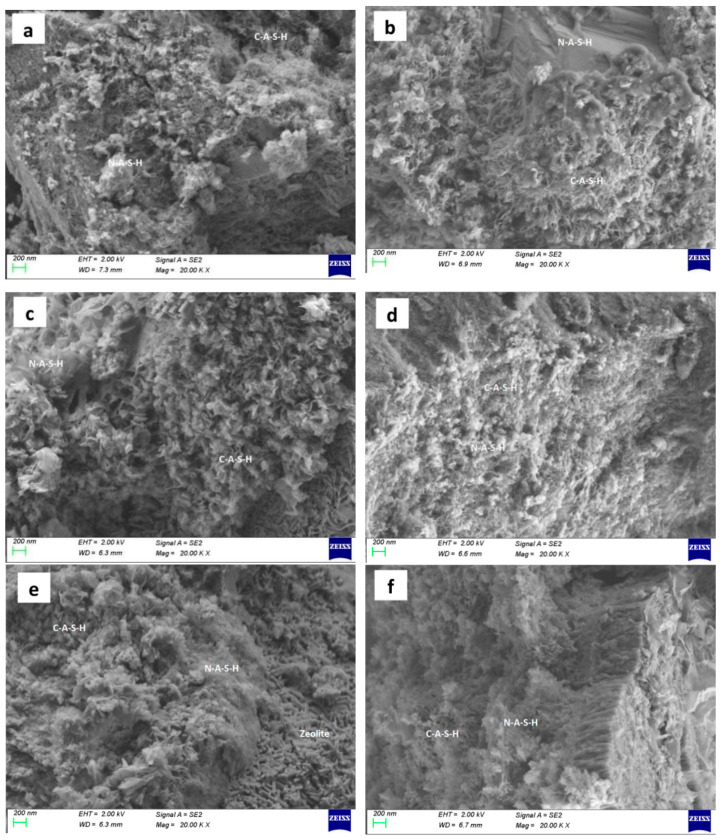

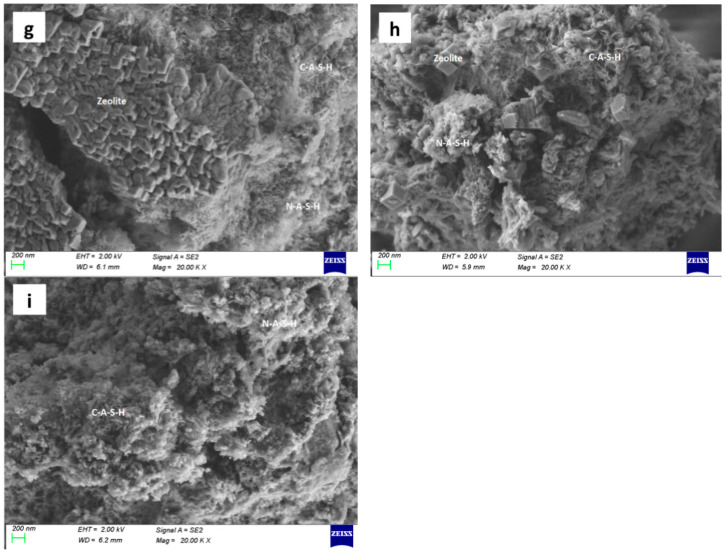
SEM of geopolymers. The magnification is20KX. ((**a**) SH (**b**) SH-water (**c**) SH-sulphate (**d**) SS (**e**) SS-water (**f**) SS-sulphate (**g**) SSG (**h**) SSG-water (**i**) SSG-sulphate).

In Figure 18, it can be seen that C–A–S–H gel, N–A–S–H gel, and C–N–A–S–H gel were formed in the SH, the SS, and the SSG geopolymers. The morphology of geopolymer changed after W-D cycles. The change of shrinkage performance and morphology of geopolymer in a dry environment were caused by the rearrangement and redistribution of C–(A)–S–H, N–A–S–H and C–N–A–S–H nanoparticles over time. The gelled material was more likely to collapse and redistribute after drying, and part of the initial large gel pores was restructured or transformed into smaller pores [20]. This corresponded to the previous conversion from blue to white for denser ceramic materials, as shown in Figure 3, Figure 4, Figure 5 and Figure 6. Moreover, this change was an irreversible process, resulting in a decrease in strength with the increase of drying times, which corresponded to the decrease of geopolymer strength after the previous W-D cycles.

Radially shaped and clumped C-A-S-H and N-A-S-H gels are in the SH geopolymer visible in Figure 18a. In Figure 18b,c, after the W-D cycles of the SH geopolymer, the clumped cement-like material was dispersed and collapsed, forming multiple microscopic pores after rearrangement. Figure 18d shows large pieces of dense gel connected in SS geopolymer. Many rod-like zeolite materials could be seen in the SS geopolymer after water W-D cycles (Figure 18e). Geopolymers exhibited stratification after W-D cycles in sulphate solution (Figure 18f). The strength of geopolymer is related to the amount of gel material and zeolite produced. If there are many cementing substances and zeolite generated in the polymerization, the strength of the specimen will be high. SS geopolymer had a large area of interwoven and dense C–A–S–H and N–A–S–H gels and zeolite, so its strength was greater than SH. The reason for this is that the SS alkali activator was Na_2_SiO_3_ and NaOH solution, Al^3+^ released faster than Si, and enough sodium silicate dissolved aluminum would react with silicon to form oligomeric aluminosilicate, forming sodium aluminosilicate hydrate (N–A–S–H, C–N–A–S–H) gels and zeolites [33].

Figure 18g shows the distribution and arrangement of large flaky zeolite minerals in the SSG geopolymer. The geopolymer had high strength after 28 days of curing under standard curing conditions. However, in W-D cycles, the addition of desulfurized gypsum promoted the exchange and combination of a large amount of Ca^2+^ and Na^+^, and the material forms were rearranged to generate Ca-rich and other C–N–A–S–H cementing materials, forming a network and large agglomerated morphology (Figure 18h,i). Meanwhile, a large number of voids appeared, which was reflected in the decrease in strength.

## 6. Conclusions

The compressive and flexural strength of the SH, the SS, and the SSG geopolymers under the action of W-D cycles were compared and analyzed. XRD, FTIR, and SEM were used to analyze their mechanism, and the following conclusions are drawn:

(1) The influence of dry and wet cycles on the flexural strength of geopolymers was greater than the compressive strength. The final sulphate cycle compressive strength of the SH and the SS was higher than that of the water cycle, while the sulphate corrosion resistance of the SSG was weak.
(2) In terms of compressive strength, the SS geopolymer had stronger erosion resistance than the SH. After 150 cycles of W-D cycling, the SS geopolymer’s compressive strength could still reach more than 39 MPa. In terms of flexural strength, the erosion resistance of the SH geopolymer was better than that of the SS, and the compressive strength was more than 2.5 MPa after 150 cycles. The SSG geopolymers had the weakest resistance to erosion.
(3) In the W-D cycles, the colour change, flexural resistance, and compressive strength of geopolymers were reduced because of the migration of water and the transition of polymerization reaction from the outside to the middle recombination. The SH recombination polymerization reaction is weaker than the SS and the SSG, resulting in fewer cracks and better bending strength. A large amount of zeolite minerals and dense gelling substances were generated in SS, such as Na-rich C–N–A–S–H, so its strength was higher. Because of the presence of desulfurized gypsum in the SS activator, a large amount of Ca^2+^ participated in the reaction to generate Ca-rich C-N–A–S–H, leading to premature external spalling and the most serious erosion.
(4) The K_f sulphate_ of the SH and the SS specimens was higher than the K_f water_, which proved that the sulphate solution promoted the hydration reaction in the sulphate cycles, and the C–N–A–S–H gel produced by the recombination reaction was greater than that of the water cycles, which effectively controlled the strength loss. Due to the rapid recombination and polymerization of SSG in the sulphate cycles, the outer skin was detached prematurely from the internal specimen, and the internal specimen was brittle and lost strength under the influence of sulphate crystallization.

## Figures and Tables

**Table 1:** Comparison table of various factors of geopolymers.

Type	Geopolymer	Cracks	Polycondensation	Cycles	Damage or Ultimate Compressive/Flexural Strength (MPa)	K_f_
Water cycles	SH	*	*	150	24/2.6	0.79
	SS	**	**	150	39.6/1.1	0.95
	SSG	***	**	20	32.2/0	-
Sulphate cycles	SH	*	*	150	26.7/2.5	0.88
	SS	**	**	150	40.8/1.1	0.98
	SSG	***	**	3	9.7/0	-

Cracks: Not easy to generate cracks *, easy to generate cracks **, rapid to generate cracks ***. Polycondensation: whiter geopolymers **, less white geopolymer *.

## Data Availability

The data presented in this study are available on the request from the corresponding author.
